# Safety and efficacy of monthly high-dose vitamin D_3_ supplementation in children and adolescents with sickle cell disease

**DOI:** 10.1007/s00431-024-05572-w

**Published:** 2024-05-14

**Authors:** Diana Hanna, Doaa E. Kamal, Hebatallah M. Fawzy, Reham Abd Elkhalek

**Affiliations:** 1 Pediatric Hematology and Oncology Unit, Pediatrics Department, Faculty of Medicine, Zagazig University, Zagazig, Egypt; 2 Rheumatology and Rehabilitation Department, Faculty of Medicine, Zagazig University, Zagazig, Egypt; 3 Public Health and Community Medicine Department, Faculty of Medicine, Zagazig University, Zagazig, Egypt

**Keywords:** Vitamin D supplementation, Sickle cell disease (SCD), Hand grip strength (HGS), Health-related quality of life (HRQL)

## Abstract

Little is known about the impact of vitamin D supplementation on hand grip strength (HGS) and health-related quality of life (HRQoL) in children and adolescents with sickle cell disease (SCD). We aimed to evaluate the safety and efficacy of monthly high-dose vitamin D_3_ supplementation and its implications on bone mineral density (BMD), HGS, and HRQoL in patients with SCD and healthy controls. The study included 42 children with SCD and 42 healthy matched controls. The study participants were supplemented with high-dose monthly oral vitamin D_3_. Changes in the serum level of 25(OH) vitamin D_3_, maximum HGS, and BMD from baseline to 6 months were assessed, and the HRQoL questionnaire and Childhood Health Assessment Questionnaire (CHAQ) were used to evaluate the functional capacity. At baseline, SCD subjects had poorer growth status indicated by negative *Z* scores. Suboptimal BMD was detected by significantly lower *Z* score, and lower HGS and worse HRQL parameters were found compared to the controls (*P* < 0.001). Median 25(OH) vitamin D_3_ was significantly lower in SCD patients compared to controls (16.5 vs. 28 ng/mL, respectively (*P* < 0.001)). After 6 months of vitamin D supplementation, there was significant improvement in the DEXA *Z*-score (*P* < 0.001), limitation of physical health (*P* = 0.02), pain scores (*P* < 0.001), and CHAQ grades (*P* = 0.01) in SCD patients. A significant improvement in HGS (*P* < 0.001 and *P* = 0.005) as well as the CHAQ score (*P* < 0.001 and *P* = 0.003) was detected in the SCD group and controls, respectively. There were no reported clinical adverse events (AEs) or new concomitant medications (CMs) during the study duration, and safe levels of Ca and 25 (OH) D3 were observed at 3 and 6 months for both groups. There was a significant positive correlation between HGS and total physical score (*r* = 0.831, *P* < 0.001) and a negative correlation with CHAQ score (*r* =  − 0.685, *P* < 0.001). We also detected a significant positive correlation between vitamin D levels at 6 months and HGS (*r* = 0.584, *P* < 0.001), pain score (*r* = 0.446, *P* < 0.001), and a negative correlation with CHAQ score (*r* =  − 0.399, *P* < 0.001).

*Conclusion*: Monthly oral high-dose vitamin D supplementation was safe and effective in improving vitamin D levels, HGS, and HRQoL in SCD children and healthy subjects, and BMD scores in SCD patients. Further randomized controlled trials are warranted to assess an optimal dosing strategy and to investigate the impact on clinically significant outcomes in children and adolescents with SCD and their healthy counterparts.

*Trial registration*: ClinicalTrials.gov, identifier NCT06274203, date of registration: 23/02/2024, retrospectively registered.

**Table:** 

**What is known:** *• Several studies have reported a high prevalence of vitamin D deficiency and suboptimal bone mineral density (BMD) in sickle cell disease (SCD) patients.* *• Musculoskeletal dysfunction is reported in SCD patients with a negative impact on physical activity and health-related quality of life (HRQL).* *• Little is known regarding the impact of vitamin D3 supplementation in children and adolescents with SCD.*	
**What is new:** *• We found that monthly oral high-dose vitamin D3 supplementation was safe, tolerated, and effective in improving serum vitamin D levels, HGS, BMD scores, and HRQL in SCD patients.*

**What is known:**

*• Several studies have reported a high prevalence of vitamin D deficiency and suboptimal bone mineral density (BMD) in sickle cell disease (SCD) patients.*

*• Musculoskeletal dysfunction is reported in SCD patients with a negative impact on physical activity and health-related quality of life (HRQL).*

*• Little is known regarding the impact of vitamin D3 supplementation in children and adolescents with SCD.*

**What is new:**

*• We found that monthly oral high-dose vitamin D3 supplementation was safe, tolerated, and effective in improving serum vitamin D levels, HGS, BMD scores, and HRQL in SCD patients.*

## Introduction

Sickle cell disease (SCD) is a hereditary disorder characterized by chronic hemolytic anemia and vaso-occlusive crises (VOC) [1]. Musculoskeletal dysfunction is reported in SCD patients. Several factors such as anemia, VOC-related stresses (e.g., hypoxia, ischemia, oxidative stress, inflammation, and necrosis), as well as muscle microvascular remodeling, may contribute to muscle dysfunction in SCD patients [23]. As a result, the attenuated muscle strength, particularly hand grip strength (HGS) may have a negative impact on physical activity and health-related quality of life (HRQL) [3].

Several studies have reported a high prevalence of vitamin D deficiency and suboptimal bone mineral density (BMD) in SCD patients, which is linked to worse disease outcomes [4–7]. However, only a few studies reported the safety and impact of vitamin D supplementation on HGS and HRQL in pediatric SCD [8].

Handheld dynamometry is considered a valid, reliable, simple tool for the objective measurement of HGS [9, 10]. HRQL is a crucial outcome measure that provides insight into the well-being of children with SCD [11]. The Childhood Health Assessment Questionnaire (CHAQ) is commonly used to assess health status in children, and the updated versions showed improved validity in a variety of musculoskeletal problems [12, 13].

This study aims to evaluate the safety and efficacy of monthly high-dose vitamin D_3_ supplementation in patients with SCD and healthy controls and its implications on BMD, HGS, and HRQL.

## Material and methods

We enrolled 42 children with SCD (HbSS, HbSβ0 thalassemia genotype), aged ≤ 18 years old, male or female at a steady state (≥ 1 month from blood transfusion and ≥ 14 days following one of the SCD complications as hospitalization for VOC or acute chest syndrome (ACS)), stable hemoglobin (Hb) level near their usual baseline, and stable dose of hydroxyurea (mg/kg) for at least 90 days before enrollment. Eligible patients were recruited from the Pediatric Hematology outpatient clinic at Zagazig University. A control group of 42 healthy age and sex-matching children were also included. We excluded SCD patients who were on chronic blood transfusion therapy, had comorbid chronic conditions, or were on medications known to interfere with calcium or vitamin D absorption or metabolism, known hypercalcemia or vitamin D hypersensitivity, vitamin D treatment for rickets, presence of urolithiasis, liver or renal impairment, and malabsorption disorders. We also excluded obese children with body mass index (BMI) > 85th percentile for age and sex [14] as the adipose tissue is the main site for storing vitamin D [15].

The study was approved by the Institutional Review Board (IRB) of the Faculty of Medicine Zagazig University (IRB No. ZU-IRB #10584). Legal guardians signed informed written consent before participating in the study, and assent was taken from the children aged 12–18 years.

### Study design

This was an interventional study (ClinicalTrials.gov Identifier: NCT06274203, on 23/02/2024, retrospectively registered). Subjects within each group, SCD or controls, received monthly oral vitamin D_3_ doses according to the baseline status of vitamin D as follows: sufficient: 100,000 IU, insufficient: 150,000 IU, and deficient: 200,000 IU. The study was conducted throughout the duration from May 2023 to Feb 2024; the enrollment period was an average of 3 months, from 3 May 2023 to 30 July 2023, with visits at baseline, 3, and 6 months. The last subject was completed on 30 January 2024. Monthly phone calls were made to support compliance with therapy and collect any adverse events (AEs) or new concomitant medications (CMs).

#### Measured outcomes in both group

1. Serum 25(OH)D level changes from baseline at 6 months.
2. BMD score changes from baseline at 6 months.
3. HGS changes from baseline at 6 months.
4. HRQL assessed by HRQL questionnaires, change of scale from baseline at 6 months.
5. Childhood health assessment by CHAQ, change of scale from baseline at 6 months.
6. Serum concentrations of inflammatory markers (CRP and ESR) change from baseline to 6 months in the SCD group only.
7. Safety reporting of any side effects (e.g., nausea, vomiting, anorexia, constipation, cardiac arrhythmias, renal failure, drowsiness, and coma).
8. Safety measurements of serum Ca and 25(OH)D levels at 3 months.

#### Anthropometric measures

A complete physical examination was performed including anthropometric measurements [16]. We calculated the BMI from weight (kg/m^2^) using a digital scale (Scaletronix, White Plains, NY) and height using a stadiometer (Holtain, Crymych, UK). Age- and gender-specific *Z* scores for weight, height, and BMI were generated based on Centers for Disease Control and Prevention 2000 reference standards [14].

#### Assessment of HGS [17]

Initially, the preference for one hand was ascertained, then the participants warmed up by gripping the handle, adjusting their grip, and going through two to three testing trials to become acquainted with a handheld dynamometer. The American Society of Hand Therapists (ASHT) standard operating protocols are followed when taking the measurement [18]. The participants support their feet while sitting up in a chair. The arm being examined is placed on a table with the elbow in 90° of flexion, the forearm in 0° of pronation and supination, the wrist in neutral resting position, and the shoulders slightly abducted (~ 10°) and neutrally rotated [18]. Starting with the dominant hand, each participant makes three maximal voluntary contractions for each hand. For additional analysis, the three test averages are computed to two decimal places.

#### The Childhood Health Assessment Questionnaire [19]

After CHAQ was translated into Arabic, it was validated to assess the functional impairment [19]. It consists of thirty questions divided into eight categories: dressing and grooming; arising; eating; walking; hygiene; reach; grip; and activities. There are four potential responses to each question: “without any difficulty” (score 0); “with some difficulty” (score 1); “with much difficulty” (score 2); and “unable to do” (score 3). If the domain is ranked lower (0/1)m then aid assistance, equipment, or assistance from another person receives at least a score of 2. A summary score known as CHAQ‐DI, which varies from 0 to 3, is calculated by averaging the highest score in each domain. For a CHAQ-DI score to be considered minimally clinically significant, it must be ≥ 0.75.

#### HRQL questionnaire (the SF-36 v2 questionnaire) [20]

The SF-36v2 was translated into Arabic language and adapted [21]. The questionnaire was scored following standard guidelines and divided into eight subscales: physical function, role limitations resulting from physical health, bodily pain, general health perception, vitality, social function, role limitations resulting from emotional problems, and mental health [20]. For each subscale: a higher score indicated good health and ranged from 0 to 100.

#### Laboratory assessment

Serum 25(OH)D was determined quantitatively by radioimmune assay (Roch Diagnostic Mannheim, Germany). Subjects were classified based on vitamin D status into vitamin D sufficient: > 30 ng/mL, insufficient: 20–29.9 ng/mL, and deficient: < 20 ng/mL [22]. Vitamin D_3_ dose was considered unsafe if it resulted in elevated 25(OH)D > 160 ng/mL with elevated calcium (age- and sex-specific range). Routine laboratory tests as complete blood count, CRP, ESR, serum Ca, and serum ferritin were also assessed.

#### BMD measured by DEXA

BMD was assessed using DEXA scan (GE-Lunar Prodigy, Madison, MA, USA) [23]. We evaluated BMD at the posterior-anterior spine. *Z*-scores were used to interpret the results, with *Z*-scores less than − 2 SD being regarded as abnormal. A *Z*-score between − 1 and − 2 SDs was used to indicate osteopenia, whereas a *Z*-score above − 1 SD was used to define a normal BMD [24].

### Statistical analysis

Sample size calculation was done using OpenEpi program version 3 (www.OpenEpi.com). Considering the Mean ± SD of HGS among cases and controls (16.2 ± 7.9, 21.9 ± 9.9 respectively) [8], the minimum appropriate sample size (which achieve power of ≥ 80%) was calculated as 78 participants (39 patients and 39 controls). Thus, eligible 84 participants were included (divided into 2 equal groups, 42 patients and 42 controls), and the power was calculated as 83.06%. The collected data were tabulated and analyzed using IBM SPSS Statistics, version 26 (IBM; Armonk, New York, USA). Continuous quantitative variables were expressed as the mean ± SD or median and interquartile range (IQR), and categorical qualitative variables were expressed as numbers and percentages. Continuous data were checked for normality by using the Shapiro-Wilk test. Independent sample *t*-test and Mann-Whitney tests were used to compare two groups of normally and not-normally distributed data, respectively. Categorical data were compared using the chi-square test and Fisher exact test. The Spearman correlation test was used to detect the closeness of association between 2 variables. All tests were two-sided. *P*-value < 0.05 was considered statistically significant; *P*-value < 0.001 was considered highly statistically significant.

## Results

We enrolled 42 children with SCD (24 had HbSS, and 18 had HbSβ0 thalassemia genotype), 24 males, a mean age of 9.03 ± 3.7 years, and 42 healthy age and sex-matched controls. Regarding the clinical data of the SCD group, the mean disease duration was 6.6 ± 3.5, patients who experienced < 1 VOC were 21 (50%), while 50% experienced > 2 VOCs in the last 12 months. Most SCD patients (85.7%) had < 1 ACS in the last 12 months and > 2 ACS were reported in 14.3% of patients. Six (14.3%) patients received iron chelation therapy, deferasirox film-coated tablets, and all of them were compliant.

The baseline demographic variables, laboratory parameters, DEXA score, HGS, and HRQL data of SCD patients and healthy controls are presented in Table 1.

**Table 1:** Baseline characteristics of SCD patients and controls

Variable	SCD group (*n* = 42)	Control group (*n* = 42)
**Age: (years) mean ± SD**	9.03 ± 3.7	8.8 ± 3.4
**Sex: *N* (%)**		
Female:	18 (42.9%)	16 (38.1%)
Male:	24 (57.1%)	26 (61.9%)
**Weight (Kg)**	21.5 (15–27)	35 (25–45)**
**Weight (*Z* score)**	− 2.06 (− 2.7– − 1.03)	0.9 (0.2–1.3)**
**Height (cm)**	121.5 (110–140)	139 (127–155)*
**Height (*Z* score)**	− 1.05 (− 2.6– − 0.6)	0.6 (− 0.2–2.2)**
**BMI** (kg/m^2^)	15.5 (13.6–16.7)	17.1 (16.1–19.4)**
**BMI percentile**	0.67 (0.2–0.8)	72 (31–83.2)**
**BMI (*Z* score)**	− 0.74 (− 3.1–0.9)	0.58 (− 0.4–0.9)*
**Hb (g/dL)**	8.5 (7.6–9)	11.2 (10.8–12.5)**
**Reticulocytes %**	10% (6–14%)	0.6% (0.4–1.0%)**
WBCs *10^**9**^**/L**	6.9 (5.4–12.5)	6 (5.1–6.3)*
Platelets *10^**3**^** /mL**	356 (288–484)	350 (280–440)
**ESR (mm/h)**	12.5 (9–25)	10 (6–13)*
**CRP (mg/L)**	2.5 (1–5)	1.8 (1–4)
**Calcium (mmol/L)**	2.3 (2.2–2.4)	2.4 (2.3–2.5)
**Ferritin (ng/mL)**	202.5 (130–352)	37 (28–60)**
**25(OH)D** (**ng/mL)**	16.5 (9–25)	28 (19–36)**
**DEXA (*Z* score)**	− 1.5 (− 2– − 1.2)	− 1 (− 1.3– − 0.8)**
**HGS (Kg)**	8.7 (6–12)	31 (25–37.5)**
**CHAQ**	1.06 (0.6–1.8)	0 (0–0.3)**
**HRQL:**		
**Physical function**	55 (40–60)	100 (100)**
**Limitation of physical health:**	50 (50–60)	100 (90–100)**
**Emotional problem**	50 (50–60)	80 (70–100)**
**Fatigue**	50 (50–60)	100 (90–100)**
**Emotional wellbeing**	50 (40–60)	90 (90–100)**
**Social**	40 (40–50)	80 (80–100)**
**Pain**	40 (30–50)	100 (90–100)**
**General health**	40 (40–50)	100 (90–100)**
**Total physical**	48.7 (42.5–52.5)	97.5 (90–100)**
**Total mental**	51.2 (50–55)	95 (90–97.5)**

Data expressed as median (IQR)

*SCD* sickle cell disease, *BMI* body mass index, *Hb* hemoglobin, *WBCs* white blood cells, *ESR* erythrocyte sedimentation rate, *CRP* C reactive protein, *25(OH)D* 25-hydroxyvitamin D, *DEXA* dual-energy x-ray absorptiometry, *HGS* hand grip strength, *CHAQ* Childhood Health Assessment Questionnaire, *HRQL* health-related quality of life

*Significant difference (*P* < 0.05); **High-significant difference (*P* < 0.001)

**Fig. 1:**
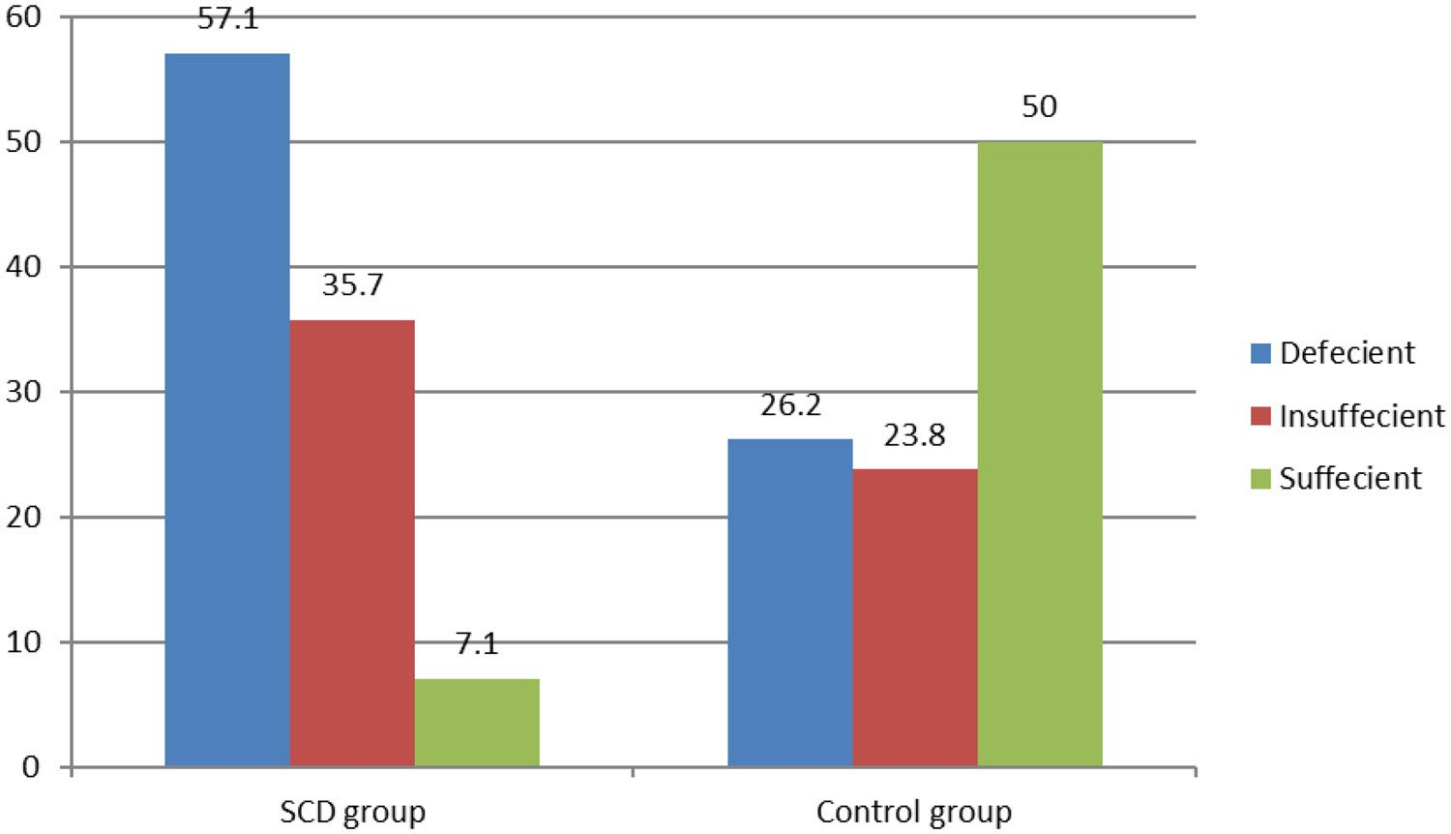
Vitamin D status among the studied groups

SCD patients had poorer growth status as indicated by negative *Z* scores for weight, height, and BMI (*P* < 0.001). Significantly higher WBCs, ESR, and serum ferritin levels were detected in the SCD group compared to controls. Suboptimal BMD was detected in SCD patients as indicated by significantly lower *Z* score compared to controls, and 4 SCD patients had a history of multiple fractures. We observed significantly lower HGS and worse HRQL parameters in the SCD group. The median 25(OH)D at baseline was significantly lower in SCD patients when compared to controls (16.5 vs. 28 ng/mL respectively). Vitamin D status in SCD and controls is shown in Fig. 1.

All SCD patients and 33 healthy controls completed the study (9 controls lost follow-up). Monthly oral high doses of vitamin D improved vitamin D levels at 6 months in both SCD and control groups (*P* < 0.001). After 6 months of vitamin D supplementation, we detected a significant improvement in the DEXA *Z* score (*P* < 0.001), limitation of physical health (*P* = 0.02), emotional wellbeing (*P* < 0.001), pain scores (*P* < 0.001), and CHAQ grades (*P* = 0.01) in SCD patients. A significant improvement in HGS (*P* < 0.001 and *P* = 0.005) as well as the CHAQ score (*P* < 0.001 and *P* = 0.003) was detected in the SCD group and controls, respectively. A significant decrease in ESR was observed in SCD patients at 6 months (*P* = 0.01) as shown in Table 2.

**Table 2:** Subjects characteristics at baseline and after 6 months of vitamin D3 supplementation

Variable	SCD		Controls	
	Baseline (*n* = 42)	At 6 months (*n* = 42)	Baseline (*n* = 42)	At 6 months (*n* = 33)
**25(OH)D (ng/mL)**	16.5 (9–25)	30.5 (24–36) **	28 (19–36)	45 (37–51) **
**DEXA (*Z* score)**	− 1.5 (− 2– − 1.2)	− 1.4 (− 1.7– − 1.2)**	− 1 (− 1.3– − 0.8)	− 0.9 (− 1.3– − 0.8)
**HGS (Kg)**	8.7 (6–12)	14.6 (9–18.4)**	31 (25–37.5)	33 (26–37)*
**CHAQ**	1.06 (0.6–1.8)	0.8 (0.4–1.7)**	0 (0–0.3)	0 (0– − 0.1)*
**Physical function**	55 (40–60)	55 (30–70)	100 (90–100)	100
**Limitation of physical health**	50 (50–60)	40 (30–60)*	100 (90–100)	90 (80–100)
**Emotional problem**	50 (50–60)	40 (30–70)	60 (60–80)	100 (90–100)*
**Fatigue**	50 (50–60)	40 (30–70)	100 (90–100)	100 (90–100)
**Emotional wellbeing**	50 (40–60)	45 (30–50)*	90 (90–100)	100 (90–100)
**Social**	40 (40–50)	35 (30–60)	80 (80–100)	100 (85–100)*
**Pain**	40 (30–50)	60 (40–70)**	100 (90–100)	100 (90–100)
**General health**	40 (40–50)	40 (30–60)	100 (90–100)	100 (90–100)
**Total physical**	48.7 (42.5–52.5)	41.2 (30–60)	97.5 (90–100)	95 (90–100)*
**Total mental**	51.2 (50–55)	40 (30–60)	95 (90–97.5)	95 (90–97.5)
**ESR (mm/h)**	12.5 (9–25)	8 (5–16)*	10 (6–13)	9 (6–12)
**CRP (mg/L)**	2.5 (1–5)	2.3 (1–4.5)	1.8 (1–4)	1.7 (1.4)
**CHAQ grade:**				
Normal	6 (14.3%)	12 (28.6%)*	27 (64.3%)	23 (69.7%)
Mild	12 (28.6%)	9 (21.4%)	15 (35.7%)	10 (30.3%)
Mild to moderate	9 (21.4%)	9 (21.4%)	0 (0%)	0 (0%)
Moderate	9 (21.4%)	12 (28.6%)	0 (0%)	0 (0%)
Severe	6 (14.3%)	0 (0%)	0 (0%)	0 (0%)

Data expressed as median (IQR). Test: chi-square for trend

*Significant difference (*P* < 0.05); **High-significant difference (*P* < 0.001)

Vitamin D levels at baseline and after 6 months among the studied groups (sufficient, insufficient, and deficient) in SCD and controls are presented in Table 3.

**Table 3:** Vitamin D levels at baseline and after 6 months among the studied groups

Variable	Baseline	At 6 months
**SCD group:**		
Deficient (*n* = 24)	10 (8–14.7)	25.5 (20.5–34)**
Insufficient (*n* = 15)	25 (22–26)	33 (30–36)*
Sufficient (*n* = 3)	32	43
**Control group:**		
Deficient (*n* = 11)	19 (18–19)	41 (36–48)*
Insufficient (*n* = 10)	26.5 (22–28)	40.5 (32.5–60)*
Sufficient (*n* = 12)	36 (31.5–40)	48 (41.2–58)*

Data expressed as median (IQR)

*Significant difference (*P* < 0.05); **High-significant difference (*P* < 0.001)

The estimated compliance to vitamin D supplementation was 90% and 87%, in SCD and controls, respectively. There were no reported clinical AEs, or new CMs during the study duration, and safe levels of Ca and 25 (OH) D were observed at 3- and 6-month visits for both groups. At baseline, 34 out of 42 SCD patients (81%) were on hydroxyurea, and none of them changed the dose or status (on/off hydroxyurea) throughout the 6-month intervention.

**Fig. 2:**
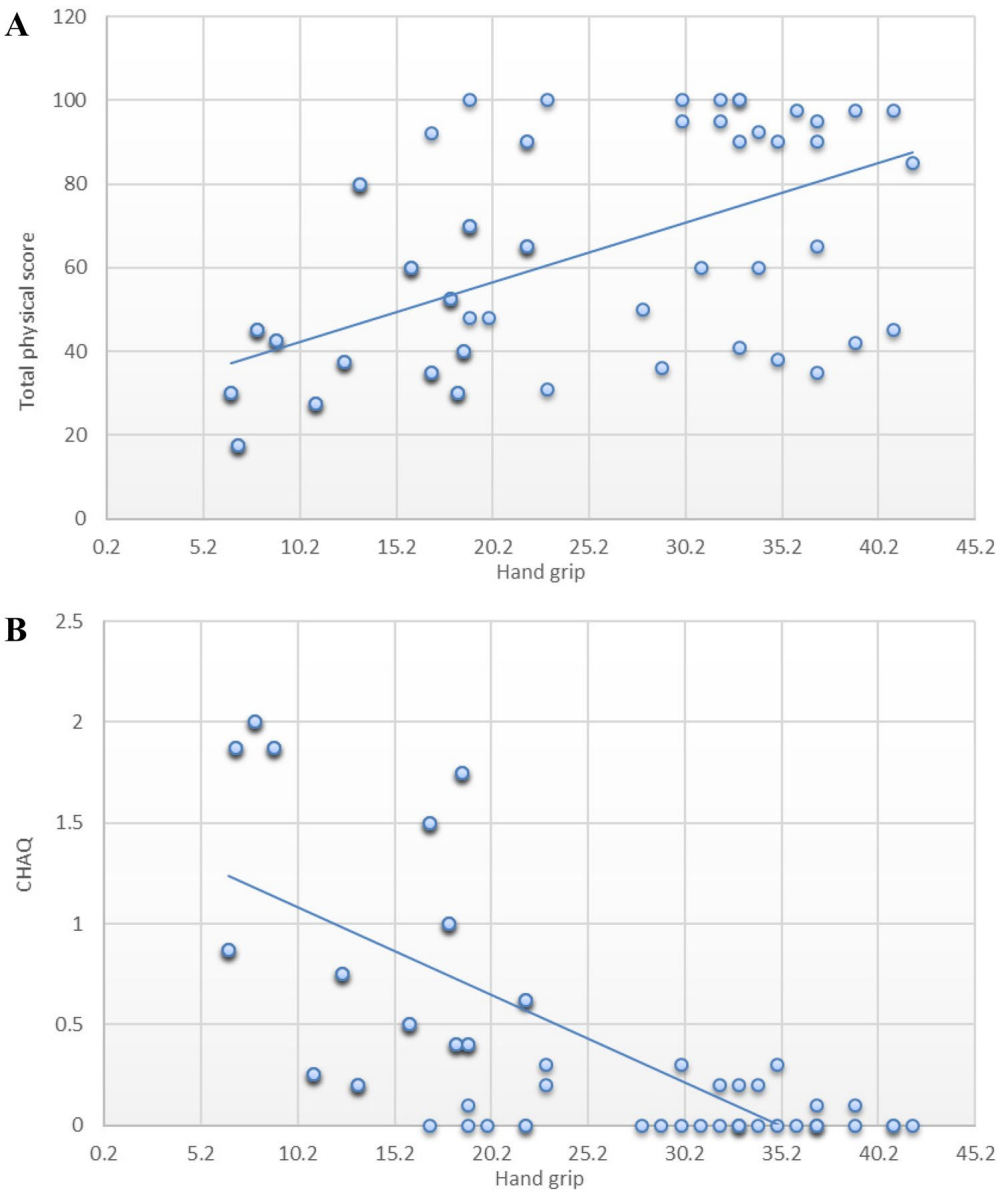
Correlation between HGS and total physical score (**A**) and CHAQ (**B**) among the studied groups

There was a significant positive correlation between HGS and total physical score (*r* = 0.831, *P* < 0.001) and a negative correlation with CHAQ score (*r* =  − 0.685, *P* < 0.001) (Fig. 2).

**Fig. 3:**
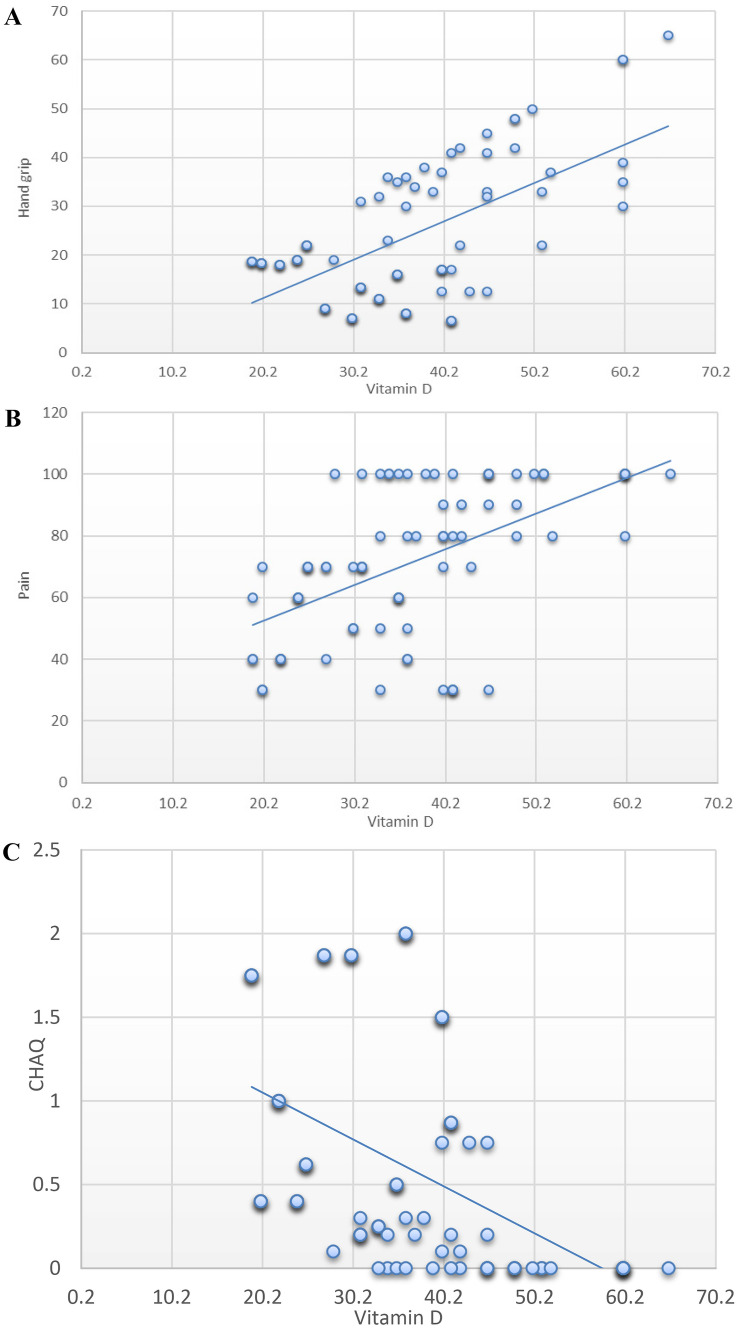
Correlation between vitamin D level at 6 months and HGS (**A**), pain (**B**), and CHAQ (**C**) among the studied groups

We also detected a significant positive correlation between vitamin D levels at 6 months and HGS (*r* = 0.584, *P* < 0.001), pain score (*r* = 0.446, *P* < 0.001), and a negative correlation with CHAQ score (*r* =  − 0.399, *P* < 0.001) (Fig. 3).

## Discussion

We detected suboptimal levels of 25(OH)D in SCD (HbSS, HbSβ0 thalassemia genotype) and healthy controls with significantly worse status in SCD patients. Combined groups at baseline, 41.65% had deficient vitamin D status, 29.75% had insufficient status, and 28.5% had sufficient levels. Moreover, suboptimal BMD was detected in SCD patients, and 4 patients reported a history of multiple bone fractures. Monthly high-dose vitamin D supplementation of 200,000, 150,000, and 100,000 IU for deficient, insufficient, and sufficient status, respectively, was safe, well tolerated, and associated with higher vitamin D levels at 6-month assessment. All groups succeeded in restoring sufficient vitamin D status except the deficient SCD group with a median level of 25.5 (20.5–34) at 6-month post-intervention; however, there was a highly significant improvement from baseline levels (*P* < 0.001). This high-dose regimen also led to a significant improvement in BMD in the SCD group, defined by the DEXA *Z* scores (*P* < 0.001). It is worth mentioning that our study was not randomized, blinded, or placebo-controlled as we considered giving a placebo for vitamin D-deficient subjects for 6 months was not ethical.

In line with these findings, high vitamin D doses (240,000 to 600,000 IU) given over 6 weeks in a pilot study were reported to be safe and effective in normalizing vitamin D status [25]. However, pre- and post-intervention BMD was not assessed. A meta-analysis by Brustad et al. concluded that high doses of vitamin D (daily doses to 10,000 IU/d or bolus doses to 600,000 IU) were safe with no increased risk of SAEs in young children aged 0 to 6 years [26]. Williams et al. studied 4 SCD children with severe vitamin D deficiency, who received oral vitamin D_3_ 100,000 IU EOW for 8 weeks followed by monthly 100,000 IU for 22 months which improved vitamin D deficiency and BMD scores with no reported AEs [27]. Another study showed that monthly oral doses of vitamin D with 100,000 or 12,000 IU for 2 years improved respiratory disease rates > 50% in SCD children aged 3 − 20 years [28]. Dougherty et al. reported that daily supplementation of vitamin D_3_ with a high dose of either 4000 or 7000 IU for 12 weeks was efficacious and safe in both HbSS patients and healthy children [29]. A recent randomized controlled trial reported that a daily dose of 1000 IU vitamin D_3_ and a high-dose vitamin D bolus will maintain 25(OH)D levels ≥ 75 nmol/L in SCD patients, however, 64 AEs were reported in 28 participants [30]. The most commonly reported AEs were vaso-occlusive crisis, fever, cold, chronic pain, headache, small red bumps, nausea, and vomiting. However, no SAE occurred during this study [30].

Consistent with our finding, many studies have reported low BMD in 28 to 64% of pediatric SCD patients [4, 31], and many of these patients were found to be vitamin D deficient (< 12 ng/mL) [4, 32]. However, long-term studies on vitamin D supplements relating to bone mineralization in SCD patients are still required.

Deficient muscle strength was reported in SCD children compared to controls with a negative impact on HRQL [2, 29, 33, 34]. In this study, we observed a significantly lower HGS and poorer HRQL parameters in the SCD group (*P* < 0.001), a significant positive correlation between HGS and total physical score, and a negative correlation with CHAQ score collectively in SCD and healthy subjects. After 6 months of vitamin D supplementation, we found a significant improvement in HGS for both children with SCD and controls (*P* < 0.001 and *P* = 0.005, respectively). This was associated with improvement in CHAQ grade, pain, physical health, and emotional well-being in SCD patients, and improvement in emotional, social, and total physical function in healthy controls. Moreover, we demonstrated a significant positive correlation between vitamin D level at 6 months and HGS and pain scores, and a negative correlation with CHAQ scores which indicate less pain and better health.

High doses of vitamin D may contribute to the enhancement of the muscular and physical function of children with and without chronic disease. Bartoszewska et al. described the molecular mechanisms of vitamin D function in muscle tissue via two pathways, the genomic pathway acts via gene transcription impacting the transportation of calcium in muscles as well as the metabolism of phospholipids, and the non-genomic pathway which controls the intracellular calcium transport stimulating the growth and proliferation of the muscle cell [35]. Dougherty et al. found that vitamin D_3_ supplementation improved muscular strength and torque in both HbSS and healthy children [8].

Pain is a hallmark of SCD with a negative impact on patient outcomes and HRQL [36–39]. A meta-analysis by Yong et al. found that vitamin D has been proven to reduce pain in patients with widespread chronic pain [40]. Osunkwo et al. performed a randomized, double-blind pilot study, in which SCD patients received either high-dose vitamin D_3_ (40,000 to 100,000 units weekly) or placebo for 6 weeks. Fewer pain days, higher quality of life scores, and higher levels of serum vitamin D were reported in the treatment group [25]. Consistent with this finding, Dougherty et al. reported a significant decrease in pain as well as fatigue, and higher HRQL in pediatric SCD patients who received high-dose vitamin D; however, they highlighted the need for further longitudinal study to detect the sustained impact with longer-term supplementation [8]. Adly et al. conducted a study on 50 children and adolescents with SCD and detected statistically lower frequencies of joint and bone pain, and sickle crisis after 3 months of vitamin D supplementation [41].

The exact mechanisms by which vitamin D supplementation lowers pain remain unclear. Vitamin D deficiency may exaggerate the disease course and aggravate the risk of complications through modification of neural and immune processes that contribute to pain perception [42]. Hood et al. reported that vitamin D supplementation to a sufficient level is one complementary therapy to decrease pain-related emergency department visits [43].

As concerns the inflammatory status in SCD patients, we detected higher ESR at baseline compared to controls. Moreover, reduced ESR levels were detected in the SCD group after 6 months of vitamin D intervention. Lee et al. reported that vitamin D supplementation affected numerous immune and inflammatory markers for SCD, including IL2, serpin E1, IFNγ, TNFα, sICAM1, and hsCRP, especially with high-dose vitamin D_3_ [44]. More studies are needed to investigate the immunomodulatory properties of vitamin D, with variable responses to different doses in SCD patients.

## Conclusion

Monthly oral high-dose vitamin D supplementation was safe, tolerated, and associated with higher vitamin D levels, improved HGS, and HRQL in both SCD children and healthy subjects as well as improved BMD scores in SCD patients. However, several enquiries remain regarding vitamin D supplementation in SCD, related to the optimal dose, duration of supplementation, long-term AEs, and efficacy in different types of SCD. Further full-scale randomized controlled trials are required to formulate standardized guidelines for optimal dosing and to investigate the impact on clinically significant outcomes in children and adolescents with SCD and their healthy counterparts.

## Limitations

The small sample size and being a nonrandomized open-label trial may limit the generalizability of our outcomes.

## Data Availability

No datasets were generated or analyzed during the current study.

## Abbreviations

ACS: Acute chest syndrome
AEs: Adverse events
ASHT: The American Society of Hand Therapists
BMD: Bone mineral density
BMI: Body mass index
CHAQ: Childhood Health Assessment Questionnaire
CMs: Concomitant medications
Hb: Hemoglobin
HGS: Hand grip strength
HRQoL: Health-related quality of life
IQR: Interquartile range
IRB: Institutional Review Board
SCD: Sickle cell disease
VOC: Vaso-occlusive crises
